# Experimental Construction of BMP2 and VEGF Gene Modified Tissue Engineering Bone *in Vitro*

**DOI:** 10.3390/ijms12031744

**Published:** 2011-03-07

**Authors:** Jia Jiang, Cun-Yi Fan, Bing-Fang Zeng

**Affiliations:** 1 Department of Sports Medicine and Arthroscopic Surgery, Huashan Hospital, Fudan University, Shanghai 200040, China; E-Mail: jessicajj19@hotmail.com; 2 Department of Orthopedics, The Sixth Affiliated People’s Hospital, Shanghai Jiao Tong University, Shanghai 200233, China; E-Mail: fancunyi888@hotmail.com

**Keywords:** bone morphogenetic protein 2, co-transfection, lentiviral vector, tissue engineering bone, vascular endothelial growth factor, β-tricalcium phosphate (β-TCP)

## Abstract

The purpose of this study was to investigate the feasibility and advantages of constructing a novel tissue engineering bone, using β-tricalcium phosphate (β-TCP) and rat bone marrow mesenchymal stem cells (MSCs), modified with human bone morphogenetic protein 2 gene (hBMP2) and human vascular endothelial growth factor 165 gene (hVEGF165), through lentiviral transfection. Both genes were successfully co-expressed in the co-transfection group for up to eight weeks confirmed by enzyme-linked immunosorbent assay (ELISA). After seeding MSCs onto the scaffolds, scanning electron microscopy (SEM) observation showed that MSCs grew and proliferated well in co-transfection group at 7 and 14 days. There was no significant difference among all the groups in hoechst DNA assay for cell proliferation for 14 days after cell seeding (P > 0.05), but the highest alkaline phosphatase (ALP) activity was observed in the co-transfection group at 14 days after cell seeding (p < 0.01). These results demonstrated that it was advantageous to construct tissue engineering bone using β-TCP combined with MSCs lentivirally co-transfected with BMP2 and VEGF165, providing an innovative way for treating bone defects.

## 1. Introduction

As an integration of *ex vivo* gene therapy and tissue engineering approach, gene modified tissue engineered bone has been recently reported to be an attractive option to treat bone defects [1–3]. Many of these studies have focused on bone morphogenetic protein 2 (BMP2) [4–6], which has the highest osteoinduction activity *in vivo* among the BMP family members [7]. In addition, vascular endothelial growth factor (VEGF), the most potent angiogenic growth factor, has also been specially chosen for several studies because vascularization appears to be a key factor in bone tissue engineering, especially for large and critical size bone defects [8–9].

Since both BMP2 and VEGF are involved in bone regeneration as osteogenic and angiogenic factors, it is possible that combined gene therapy of both genes in bone tissue engineering might have more significant effect on bone regeneration than single gene alone. Furthermore, there is concern that a single exposure to an exogenous growth factor may not induce an adequate osteogenic signal in many clinical situations with relatively limited bone healing potential because of compromised vascularity, limited bone stock, and abundant fibrous tissue [10].

To address these important questions, we constructed tissue engineering bone using β-tricalcium Phosphate (β-TCP) combined with rat bone marrow mesenchymal stem cells (MSCs) lentivirally co-transfected with human BMP2 gene (hBMP2) and human VEGF165 gene (hVEGF165), which had not been previously experimentally demonstrated. Lentiviral vector mediated transfer system was applied by virtue of its several advantages, such as high transfection efficiency, low toxicity and the ability to incorporate into the host genome allowing for prolonged target gene expression [11–12]. The feasibility of this co-transfection approach is supported by former combined gene therapy through co-transfection of other genes [13–14]. Cell proliferation and osteogenic differentiation on the scaffolds were evaluated by scanning electron microscopy (SEM) observation, hoechst DNA assay and alkaline phosphatase (ALP) activity assay.

## 2. Results and Discussion

### 2.1. ELISA

**Figure 1.**
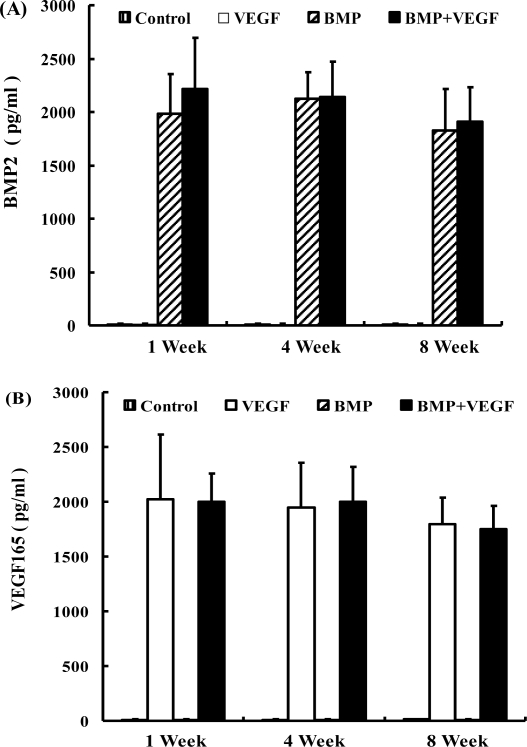
Supernatants of MSCs in each group (Control group, BMP group, VEGF group and BMP+VEGF group) were collected at 1, 4, and 8 weeks after transfection and measured with ELISA kits specific for BMP2 **(A)** and VEGF165 **(B)**. Results are shown as mean ±SD (n = 4 for each group).

In BMP+VEGF group, consistently high production of BMP2 and VEGF165 proteins was achieved for over eight weeks (Figure 1). At each time point, there was no significant difference in the amount of BMP2 production between BMP group and BMP+VEGF group (P > 0.05), whereas no detectable BMP2 was produced in control group and VEGF group. Similarly, there was no significant difference in the amount of VEGF165 production between VEGF group and BMP+VEGF group at each time point (P > 0.05), whereas no detectable VEGF165 was produced in control group and BMP group.

In recent years, *ex vivo* gene therapy of MSCs genetically modified with BMP2 has emerged as an effective strategy for bone regeneration [5,15]. Although BMP2 gene therapy is still being investigated for its therapeutic potential, a combination of BMP2 and other growth factors may prove to be a more effective initiator of bone formation. Because bone formation requires angiogenesis within newly formed tissue, it is possible that up-regulation of VEGF may also improve the efficacy of BMP gene therapy for bone regeneration. In a study by Peng, *ex vivo* gene therapy, using muscle-derived stem cell mediated delivery of VEGF in combination with BMP-4 over-expression, induced more bone formation than either of the two genes individually [8].

There are several choices of vectors for *ex vivo* gene transfer, such as plasmids, liposomes, retrovirus, adenovirus and adeno-associated virus [16]. To date, viruses are the most efficient vectors for gene delivery [17]. However, the use of these viral vectors has several disadvantages. For example, retroviral vectors require actively dividing cells for integration of the foreign gene. Transduction with adenoviral vectors is generally less efficient and requires very high vector doses [18] and the transient protein production may not produce an adequate osteogenic response in severely compromised biological environments [19]. An ideal vector would have a high transfection efficiency, low toxicity, and consistent gene expression. Recently, lentiviral vectors based *ex vivo* gene therapy has been developed for bone regeneration [17,20]. Lentiviral vectors can transduce both dividing and nondividing cells and incorporate into the host genome, allowing for prolonged target gene expression [12]. In McMahon *et al.*’s study, HIV-based lentivirus proved to be the most effective method with transduction efficiencies of up to 95%, concurrent with low levels of cell toxicity compared with adenovirus, adeno-associated virus, and nonviral vectors at delivering GFP reporter gene into rat MSCs [11].

In our study, rat MSCs infected with Lv-GFP demonstrated over 90% transduction efficiency, which was in accordance with the result in McMahon *et al.*’s study. And the hoechst assay results showed that the growth curves of all the groups were similar, indicating that lentivirus-mediated co-transferetion did not inhibit cell proliferation. ELISA results demonstrated that VEGF165 and BMP2 genes were successfully co-expressed with significant quantities for eight weeks in BMP+VEGF group, suggesting that the lentiviral vectors stably incorporated into the host genome and were passed on to all progeny cells in an expanding BMSCs population, leading to a long-term stable expression of exogenous gene rather than a single bolus or limited expression in only one generation. Prolonged gene expression may be necessary when developing tissue engineering strategies to repair large bone defects especially with a compromised host environment that is often seen in tumor resection, fracture nonunion, and revision total joint arthroplasty [17,20].

### 2.2. SEM Examination

**Figure 2.**
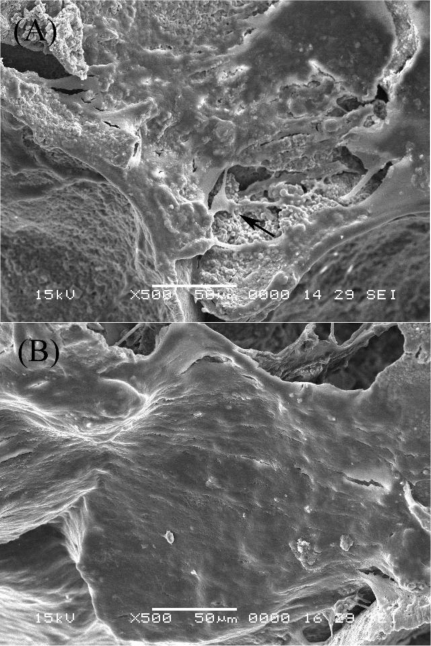
SEM images of MSCs on the β-TCP scaffolds **(A)** 3 days and **(B)** 7 days after cell seeding (Bar = 300 μm).

The morphology of MSCs co-transfected with BMP+VEGF on the scaffolds was observed by SEM at 7 and 14 days after cell seeding. At day 7, the cells adhered and differentiated well on the β-TCP scaffold with a good proliferating capability and some typical osteoblast-like cells could be seen (Figure 2A). At day 14, it was observed that most of the pore surface was covered by cells together with numerous extracellular matrices (ECM) (Figure 2B).

Bone tissue engineering has been developed as the most researched alternative to conventional therapies for fracture nonunion, spinal fusion and segmental skeletal defects following tumor resection or trauma [21–23]. Typically, bone tissue engineering methods consist of three elements: a donor cell source, a three-dimensional scaffold or matrix and osteoinductive growth factors [24]. Within this method, there is the potential to genetically modify the donor cells to express the required growth factors and the donor cells also need to be delivered in a scaffold.

A suitable scaffold for bone tissue engineering is also critical for the success of any gene therapy strategy. β-TCP shows excellent osteoconductivity and biocompatibility, and its biodegradation rate could be easily controlled. The novel porous β-TCP scaffolds applied in this study has been proved to have prominent osteoconductive activity and exhibited good osteogenic activity both *in vitro* and *in vivo* with the aid of osteogenic medium in a previous study [25,26]. In our study, SEM findings confirmed that MSCs can easily adhere to, and proliferate inside, the β-TCP scaffolds. The DNA and ALP activity assay results also indicated that the novel porous β-TCP scaffolds can support the proliferation and subsequent osteogenic differentiation of rat MSCs *in vitro*.

### 2.3. Hoechst DNA Assay

**Figure 3.**
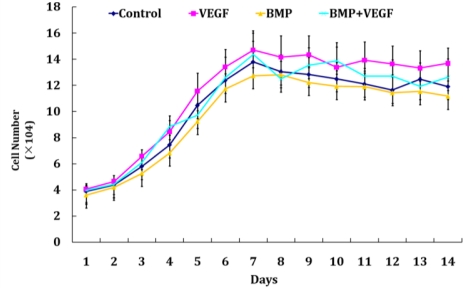
The proliferation curves of MSCs of each group (Control group, VEGF group, BMP group and BMP+VEGF group) on the β-TCP scaffolds. Cell proliferation of each group on the β-TCP scaffolds was determined every day by a hoechst assay for up to two weeks after cell seeding. Data are expressed as mean ±SD (n = 3 for each group).

Cell proliferation of MSCs for each group on the β-TCP scaffolds was determined by hoechst DNA assay for 14 days and the results are shown in Figure 3. The corresponding viable cell number of each group showed a similar trend and no significant difference could be found (P > 0.05). Viable cell number increased with time gradually during the first week of culture, peaked at day 7 or day 8 and then leveled off, following a plateau phase. However, the number of viable cells within the scaffolds still exhibited an increase of around 3-fold at day 14 compared to the initially seeding number. As for the safety profiles, the third generation HIV-1-based cloning vector system applied in our study was designed to maximize its biosafety features including the development of self-inactivating vectors producing pseudotyped lentiviral particles, minimizing the chances of generating replication-competent virus via recombination by development of a three-plasmid expression system and so on, making it possible for lentiviral-based gene therapy in preclinical models and decreasing the potential dangers of its clinical use [27,28].

### 2.4. ALP Activity Assay

**Figure 4.**
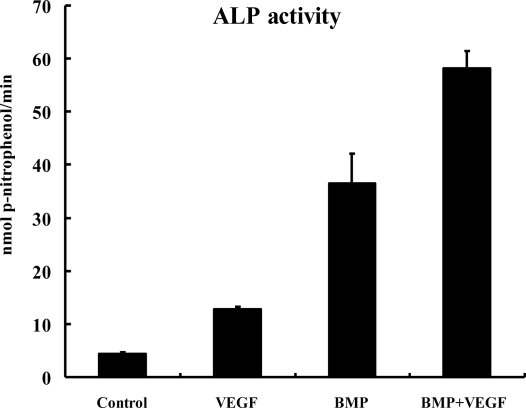
Detection of alkaline phosphatase (ALP) activity for each group (Control group, VEGF group, BMP group and BMP+VEGF group) at 14 days after cell seeding. Results are shown as mean ±SD (n = 5 for each group).

The ALP activity result at 14 days after cell seeding is shown in Figure 4. The ALP activity was stimulated by 2.9-fold in VEGF group compared to that in control group (P < 0.01). In BMP group, the ALP activity was further stimulated by 8.3-fold compared to that in control group (P < 0.01); and the ALP activity was 1.6-fold greater in BMP+VEGF group compared to that in BMP group (P < 0.01).

ALP has been implicated as a marker of osteoblast differentiation which is expressed early in the process and its expression persists throughout the maturation of the osteoblast [29–31]. Gene delivery of BMP2 could augment the expression of alkaline phosphatase in BMSCs [15,32], which was in accordance with the results in our study. Liquid recombinant murine VEGF was reported to induce alkaline phosphatase activity in primary human osteoblasts [33]. Midy and Plouet noted that the alkaline phosphatase expression of fetal bovine osteoblasts was significantly increased when exposed to rhVEGF [34]. Treatment of KS483 cells (preosteoblast-like cell line) with rhVEGF-A, stimulated alkaline phosphatase nodule formation [35]. All the above research suggested that VEGF might have a direct autocrine role in osteoblast differentiation. In our study, the ALP activity was also significantly increased in MSCs infected with Lv-VEGF when compared to control group, but lower than that in BMP group. And co-expression of BMSCs with BMP2 and VEGF resulted in an increased ALP activity than the BMP group. These observations implied that the stimulation of ALP activity in the co-transfected MSCs was the result of the synergistic action of endogenous BMP2 and VEGF proteins produced by double-transfected cells. Similar research by Yeh LC and Lee JC showed that significant enhancement of osteogenic cell differentiation evaluated by ALP activity could be accomplished by co-transfection with an osteogenic protein gene, such as osteogenic protein-1(OP-1) and an enhancer gene, such as insulin-like growth factor 1 (IGF-I) [36].

## 3. Experimental Section

### 3.1. MSCs Isolation and Expansion

Rat bone marrow was obtained from the femurs and tibias of 2-week-old Wistar rats by a technique described previously [15], and cultured on tissue culture dishes (Falcon, BD Labware) in low-glucose Dulbecco’s modified Eagle’s medium (DMEM) supplemented with 10% (v/v) fetal bovine serum (FBS), 1% penicillin/streptomycin at 37 °C in 5% CO_2._ All procedures were performed at the facility accredited by the Animal Care and Experiment Committee of Shanghai Jiao Tong University School of Medicine. MSCs can adhere to the surface of culture dishes (whereas hemopoietic cells cannot), so the adherent cells were isolated from bone marrow with medium changed twice a week through adherence-separation culturing. When reaching 80% confluency, cells were detached with 0.25% (w/v) trypsin/1mM EDTA solution (1:1,v/v), replated at 1×10^4^ cells/cm^2^ on tissue culture dishes and cultured as first-passage cells (P1) until confluency (5–7 days). MSCs at passage 2 were used for transfection.

### 3.2. Lentiviral Vector Construction, Virus Production, and Infection

hBMP2 and hVEGF165 cDNAs were amplified from human osteosarcoma MG-63 cell line by polymerase chain reaction (PCR) using the primers described in Table 1. Then hBMP2 and hVEGF165 cDNAs were subcloned into the pCDH expression lentivector (System Biosciences) respectively. 293TN producer cells were co-transfected with pPACK Packaging Plasmid Mix (System Biosciences) and expression lentivector (containing hBMP2 or hVEGF165) or control green fluorescent protein (GFP) plasmid by using Lipofectamine™2000 (Invitrogen). Thus, the lentivirus containing BMP2 cDNA (Lv-BMP), lentivirus containing VEGF165 cDNA (Lv-VEGF) and lentivirus containing GFP cDNA (Lv-GFP) were obtained.

**Figure 5.**
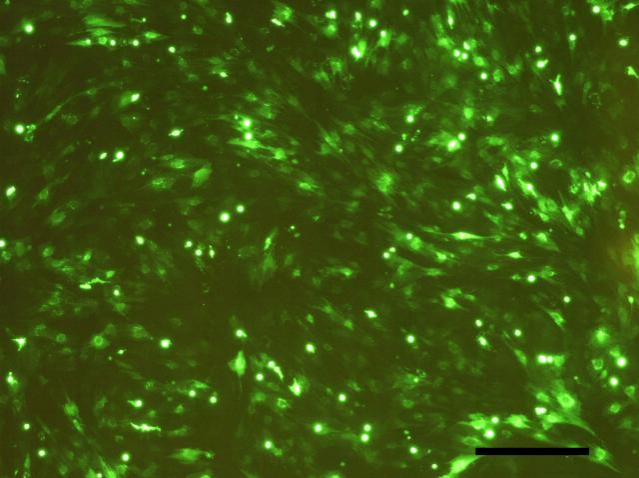
Rat MSCs of passage2 in culture were infected with Lv-GFP at a MOI of 40. A transfection efficiency up to 90% was obtained, determined by the fraction of fluorescent cells with fluorescent microscopy at 4 days after transfection (Bar = 500 μm).

Rat MSCs of passage2 in culture were infected with Lv-GFP at various multiplicity of infection (MOI) of 5, 10, 20, and 40. The efficiency of lentiviral gene transfer in MSCs was quantitatively determined by the fraction of fluorescent cells with fluorescent microscopy according to the manufacturer’s instructions (System Biosciences) at 4 days after transfection. As a result, the fraction of green-glowing cells reflecting the lentiviral gene transfer efficiency was dose dependent in the range of 5–40 MOI (data not shown). The highest transfection efficiency up to 90% was obtained at a MOI of 40 (Figure 5), while flow cytometry studies demonstrated minimal apoptosis (data not shown). There were four groups in this study: MSCs not infected with lentivirus (Control group), MSCs infected with Lv-BMP (BMP group) at 40 MOI, MSCs infected with Lv-VEGF (VEGF group) at 40 MOI, MSCs infected with Lv-BMP at 40 MOI and Lv-VEGF at 40 MOI (BMP+VEGF group). Rat MSCs of passage2 in culture were infected according to the four groups described above in the presence of 5 μg/mL polybrene (Sigma).

### 3.3. Enzyme Linked Immunoabsorbent Assay (ELISA)

The *in vitro* hBMP2 and hVEGF165 production in each group was quantified in culture supernatant at 1, 4, and 8 weeks after transduction using an ELISA kit (BMP2 ELISA kit, R&D system; VEGF165 ELISA kit, BIOSOURCE) according to the manufacturer’s instructions. The medium was changed every 24 hours during the ELISA detection period.

### 3.4. Preparation of Porous β-TCP Scaffolds and Cell Seeding

The β-TCP scaffolds with dimensions of 3 mm × 3 mm × 3 mm were kindly provided by Shanghai Tissue Engineering Research and Development Center. The scaffold porosity is 90.7 ± 1.9%, with pore sizes ranging from 300 to 500 μm. The scaffolds with dimension of 3 mm × 3 mm × 3 mm were sterilized in an autoclave before use.

MSCs of each group were collected and seeded at a density of 1 × 10^6^ in 30 μL on the β-TCP scaffolds and incubated at 37 °C for 2 h to allow cells to attach to the scaffolds. Then the scaffolds were transferred into a 96-well plate, respectively. Each well with one scaffold was cultured with the normal culture medium to form tissue engineering bone. The medium was changed every 2–3 days after cell seeding on the scaffolds.

### 3.5. Scanning Electron Microscopy (SEM)

The β-TCP scaffolds combining MSCs of BMP+VEGF group were randomly chosen for SEM observation at 7 and 14 days after cell seeding. Samples were firstly fixed with 2% parformaldehyde/2.5% glutaraldehyde in 0.1 M sodium cacodylate buffer (pH 7.4), postfixed for 48 h in 0.1% osmium tetroxide (OsO_4_), dehydrated through an ethanol series, and dried in a CO_2_ critical point dryer for 12 h. The samples were then sputter coated with gold and imaged at 15 keV with a scanning electron microscope (JSM-6360, JEOL, Japan).

### 3.6. Hoechst DNA Assay

Cell proliferation of each group was determined every day by a hoechst assay for up to two weeks after cell seeding. A DNA standard was prepared by lysing serial dilutions of a known concentration of MSCs. The amount of DNA within the scaffolds and standards was determined using Hoechst 33258 dye. Fluorescence was measured at excitation wavelength 355 nm and emission wavelength 465 nm on a fluorescence plate reader. Cell number in the scaffolds was determined by comparing sample fluorescent results to the cell standard curve.

### 3.7. ALP Activity Assay

The cell-seeded scaffolds of each group were analyzed 14 days after cell seeding as described below: The scaffolds were homogenized with 1mL Tris buffer (pH 7.4, Sigma), and sonicated. The cell lysate (0.1 mL) was mixed with 0.5 mL p-nitrophenol phosphate substrate solution (Sigma) and 0.5 mL alkaline phosphatase buffer solution (Sigma). After incubation at 37 °C for 15 min, 10 mL of 0.05N NaOH were added to stop the reaction. The production of p-nitrophenol in the presence of ALP was measured by monitoring light absorbance of the solution at 405 nm at 1 min increments. The slope of the absorbance *versus* time plot was used to calculate the ALP activity.

### 3.8. Statistical Analysis

The experimental results were expressed as mean ±SD. Student’s t-test was used to determine the statistical significance of the data obtained. Difference with P < 0.05 was considered statistically significant.

## 4. Conclusions

In conclusion, our data demonstrated that it was advantageous to construct BMP2 and VEGF modified tissue engineering bone through lentiviral transfection with a long-term stable co-expression of both genes and some aspects of enhanced osteogenic differentiation, thus having important implications for the new therapeutic strategies to enhance bone regeneration. Although both VEGF and BMP2 can stimulate osteogenic differentiation, the key advantage of local VEGF gene therapy may be its ability to couple angiogenesis with bone formation and remodeling *in vivo*. Whether the novel tissue engineering bone will enhance bone regeneration *in vivo*, is an important area of experimentation and will be further studied.

## Figures and Tables

**Table 1.** Primers of vascular endothelial growth factor 165 gene (VEGF165) and bone morphogenetic protein 2 gene (BMP2) for the polymerase chain reaction (PCR) that amplified VEGF165 and BMP2 from human osteosarcoma MG-63 cell line.

**Gene Primers**
VEGF165
5′-CGGAATTCGCCACCATGAACTTTCTGCTGTCTTGGGTGC-3′ (forward)
5′-CGCGGATCCTCACCGCCTCGGCTTGTCACATC-3′ (reverse)
BMP2
5′-GGAATTCGCCACCTGCGGTCTCCTAAAGGTC-3′ (forward)
5′-CGGGATCCTTGCTGTACTAGCGACACCCAC-3′ (reverse)
